# Cooperative parent mediated therapy for Italian children with autism spectrum disorder: a clinical experimental study in a community healthcare service in Italy

**DOI:** 10.3389/frcha.2025.1544344

**Published:** 2025-07-01

**Authors:** Alessandra Carta, Laura Casula, Salvatorica Manca, Mariangela Valentina Puci, Giuseppina Puseddu, Elisa Fucà, Giovanni Sotgiu, Stefano Vicari, Stefano Sotgiu, Giovanni Valeri

**Affiliations:** ^1^ Unit of Child Neuropsychiatry, Department of Medicine, Surgery and Pharmacy, University of Sassari, Sassari, Italy; ^2^ Child and Adolescent Neuropsychiatry Unit, Bambino Gesù Children’s Hospital, IRCCS, Rome, Italy; ^3^ Complex Operating Unit of Child Neuropsychiatry, Department of Mental Health and Addiction Services of Sassari, San Camillo, Sassari, Italy; ^4^ Clinical Epidemiology and Medical Statistics Unit, Department of Medicine, Surgery and Pharmacy, University of Sassari, Sassari, Italy; ^5^ Complex Operating Unit of Child Neuropsychiatry, Department of Mental Health of Nuoro, Local Healthcare Organization of Nuoro, Nuoro, Italy; ^6^ Life Sciences and Public Health Department, Catholic University, Rome, Italy

**Keywords:** neurodevelopmental disorders, parent-child interaction, community healthcare service, additional therapeutic effects, autism spectrum disorder

## Abstract

**Introduction:**

This Clinical Experimental Study aimed to evaluate the effectiveness of Cooperative Parent Mediated therapy (CPMT), a targeted parent-coaching program for Autism Spectrum Disorder (ASD), in Community Healthcare Service in Italy.

**Methods:**

Forty children with ASD and their parents were randomly assigned to treatment conditions: the Control group received Individual Treatment As Usual (TAU Control group); while CPMT group received weekly parent–child sessions in addition to Individual TAU. Primary blinded outcomes were 6-months post-intervention change in parent–child interaction scores. Secondary outcomes included ASD symptom severity, adaptive functioning and parental stress levels. Baseline and post-treatment evaluations, at 6 months of follow up, were performed by an independent team.

**Results:**

CPMT group showed significant add-on benefits on parent-child interactions, severity of autism symptoms, adaptive skills and parental stress level.

**Discussion:**

This study provides preliminary evidence for the effectiveness of the CPMT model also in community services, representing a further step forward in research on the implementation of therapy for ASD in community healthcare service.

## Introduction

Autism spectrum disorder (ASD) refers to a heterogeneous group of lifespan neurodevelopmental conditions, characterized by early-onset difficulties in communication and reciprocal social interaction, associated with unusually restricted as well as repetitive behaviour and/or unusual sensory interests in the environment, which may manifest as hypo- or hyper-reactivity to sensory stimuli (for example, apparent indifference to pain or temperature, adverse responses to specific sounds or textures, excessive smelling or touching of objects, or visual fascination with lights or movement) (1).

The estimated global prevalence in 2022 was 1%, with a significant reduction in practical, social, and school or work adaptive competencies (2).

In Italy, 1 in 77 children (aged 7–9 years old) has received a diagnosis of ASD (3). It is now widely recognized that the active involvement of parents in ASD interventions can have an impact on a child's development, particularly on communication and social interaction skills, daily living skills and behavior (4–10).

Many clinical practice guidelines recommend a parent-based approach (11–16).

Parent-mediated interventions (PMIs), according to Bearss' taxonomy (17), are technique-focused interventions in which the parent serves as the agent of change, while the child is the direct beneficiary, with parents involved as “mediators” of the intervention.

A recent meta-analysis, which included 51 randomized controlled trials (RCTs), found moderately significant improvements in child outcomes compared to control conditions, with an effect size of *g* = 0.55 at the end of the intervention (6). Significant improvements were observed, with effect size measured for core ASD symptoms (*g* = 0.60), maladaptive behaviors (*g* = 0.51), and language/communication skills (*g* = 0.54). In contrast, smaller gains were observed in adaptive skills (*g* = 0.23) (6).

Research indicates that the both short- and long-term benefits of PMIs on ASD core ASD symptoms may be partially attributed to changes in the way parents interact with their children (18–20) Improving the parent-child interaction style involves parents becoming more responsive and attuned to their child's cues, which leads to better communication and emotional connections. Previous research, for example, has shown that a model of parent-mediated intervention could enhance parents' synchronous responses and increase children's initiation of communication (21). Therefore, examining proximal outcomes within the parent-child relationship provides important insights into the functioning of PMIs (22, 23). However there are still limited studies on the effectiveness of PMIs that consider the role of parental interactional style and parental characteristics at baseline of interventions (10, 24).

Although the scientific literature supports the effectiveness of evidence-based interventions for ASD, most of this evidence comes from efficacy studies carried out in controlled research settings (25), while systematic research within community health services (CHS) is rarely conducted.

Indeed, several factors may contribute to the lack of alignment between interventions tested in research environments and those implemented in community based settings such as variations in the characteristics of children, parents and therapist (26, 27). There is a need to support community practitioners in adapting evidence-based approaches for interventions with children with ASD, while balancing the researchers' focus on methodological rigor with the flexibility required for outcome evaluations in community settings (28).

Findings from a recent meta-analysis have documented that few studies have examined outcomes for children with ASD, who receive routine clinical care, or “treatment as usual (TAU)”, outside the research context. Furthermore, most studies were conducted in the US (36.4%), Australia (21.2%) and the UK (18.2%), while only 6% were conducted in Italy (29).

To assess the real-world effectiveness of ASD interventions, it is essential to consider how local healthcare systems, which vary by country, impact implementation. Differences in resources, staffing, and service structures can affect outcomes, making it crucial to conduct state-specific studies. These studies would help adapt evidence-based approaches to fit local contexts, ensuring effective interventions for children with ASD across diverse settings.

In the Italian context, public child neuropsychiatry services provide free interventions. In most cases, TAU for ASD consists of ASD speech and language therapy and/or psychomotor therapy. Consequently, many families often seek target interventions independently, outside of community services (30).

To the best of our knowledge, in Italy, only one study conducted in a public service setting has shown promising results regarding the acceptability and feasibility of a parent-focused intervention model, compared to a TAU control group (31). Specifically, the Caregiver Skills Training (CST) model appears to be effective in improving parental interactional skills, parenting stress, self-efficacy, and child gestures. However, no improvements in child ASD severity were observed (31).

Therefore, further systematic studies assessing the effectiveness specifically of PMIs models in community settings are needed.

The only RCT on PMIs conducted in Italy within a research context and included in a recent meta-analysis was the one by Valeri et al. (32), which evaluated the effectiveness of the Cooperative Parent-Mediated Therapy (CPMT) model implemented in a tertiary care center (6, 32). The CPMT is a targeted parent-coaching program focused on the core symptoms of ASD and based on the Naturalistic Developmental Behavioral Interventions (NDBI) framework, with a particular emphasis on promoting cooperative interactions (32).

The study reported a moderate improvement in autistic symptoms (*g* = 0.58), as assessed by the ADOS test, in favor of the CPMT model compared to the TAU control group (32) at the end of the intervention (6, 32).

Given the importance of assessing parent-child interaction outcomes to understand how PMIs can contribute to reducing autistic symptoms (22, 23) we conducted a Clinical Experimental Study with a random allocation design, to explore the efficacy of CPMT—implemented in a community setting—on parent-child interactions. Specifically, we aimed to evaluate whether the addition of CPMT to Treatment As Usual (TAU) would provide additional benefits for parent-child interaction (primary outcome), compared to a Control Group (CG) receiving only individual TAU (TAU CG).

Furthermore, we aimed to investigate the effect of CPMT in combination with TAU on reducing core ASD symptoms, improving adaptive functioning, and reducing parental stress levels, in comparison to individual TAU alone.

We hypothesized that CPMT could provide additional benefits in promoting parent-child interaction and would be effective in reducing core ASD symptoms, improving adaptive functioning and decreasing parental stress level.

## Methods

### Study design

The study design was a rater-blinded Clinical Experimental Study conducted in an Italian Child Neuropsychiatry community service. Clinical assessment was performed at baseline and after 6 months of therapy. This study was conducted in accordance with the principles of the Declaration of Helsinki; the Sardinian regional ethical committee approved the study (Prot. PG/2022/18028) and parents provided written informed consent. The Clinical Experimental Study it has been registered on the ClinicalTrials.gov website (https://clinicaltrials.gov), with the identifier: NCT06692946.

All 40 children received the same TAU individual treatment.

The experimental group consisted of the addition of CPMT to the individual TAU treatment (CPMT group). The Control Group (CG) was composed of the individual TAU (TAU CG). Children were randomly allocated to one of the two groups. The cognitive level and degree of language impairment for participants were assessed only during the clinical evaluation at the time of diagnosis. Therefore, these assessments were not conducted by a blinded independent assessor. In regard to the language level, it was redefined only during the standardized ADOS evaluation, as specified by the test protocol.

Therapeutic allocation was shown to all stakeholders after the baseline assessment. Outcomes were assessed at baseline (T0) and after 6 months (T1) at the end of the therapy. Assessors and supervising research staff were independent from therapists and were blinded of treatment and method of the random allocation.

### Procedures

Parents and children have been recruited at the Child and Adolescent Neuropsychiatry community service of Sardinia in Italy, between October 1th 2021 and August 31th 2022, fifty children who received ASD diagnosis, based on DSM-5 criteria, were considered eligible based on the following inclusion criteria:

(a) Age between 2 and 10 years; (b) clinical diagnosis of ASD based on DSM-5 criteria (1); (c) scores above the autism spectrum cut-off on the Autism Diagnostic Observation Schedule Generic-ADOS-2; (d) absence of other major medical diagnoses (i.e., epilepsy, genetic syndromes); (e) absence of psychosocial treatment during the trial.

**Figure 1:**
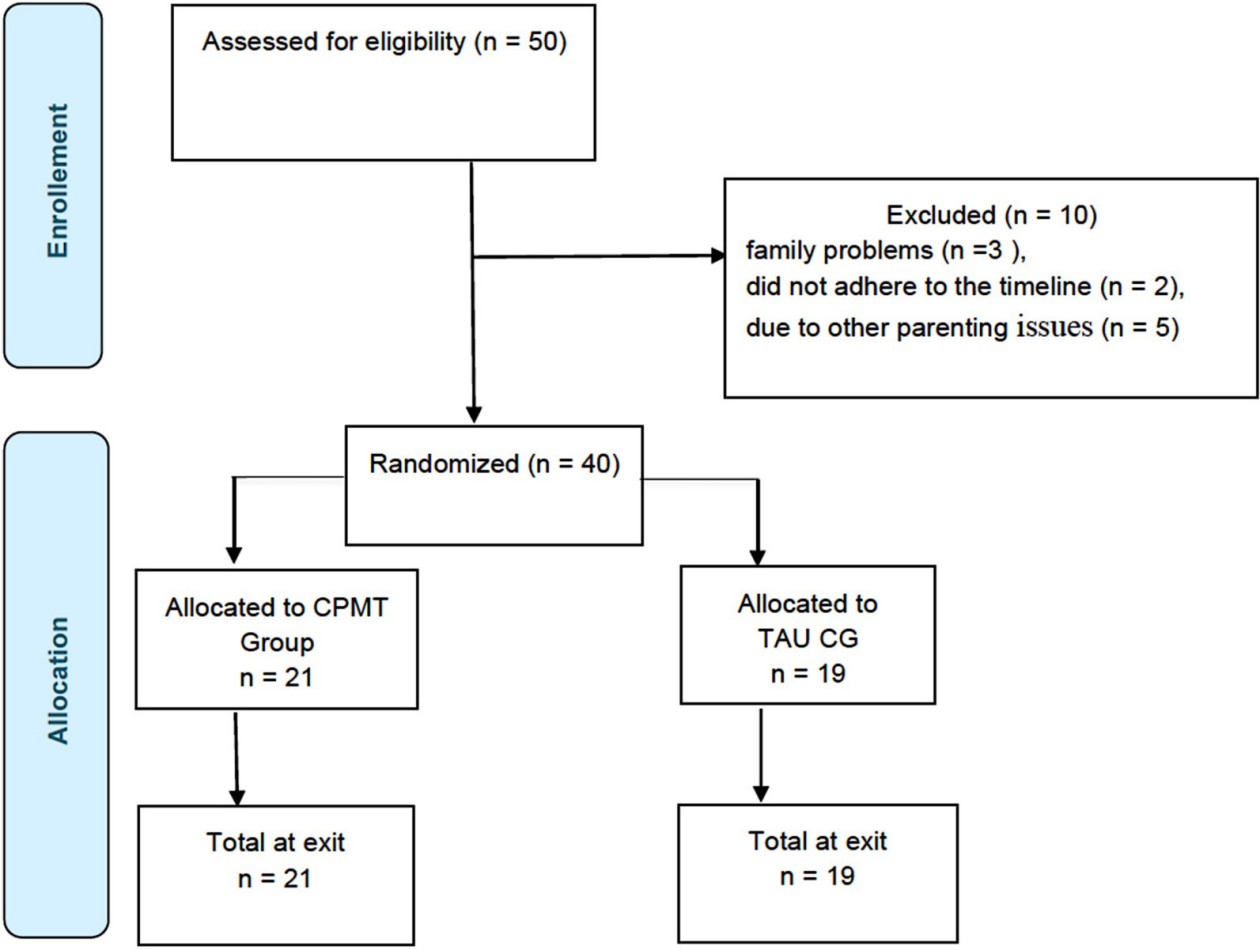
Flowchart of study enrolment, treatment, and allocation. Adapted from the CONSORT 2010 statement (63), Licensed under CC BY 4.0.

Out of 50 participants, 10 were excluded: 3 due to family problems, 2 for not adhering to the timeline, and 5 due to other parental issues (see Figure 1 enrolment flow-chart study).

The clinical evaluations were performed by a clinical team made of child neuropsychiatrists and psychologists with expertise on ASD assessment, who employed structured interviews and standardized tools to support clinical evaluation.

An independent statistician assigned an identification number to each family. After consent and baseline assessment, family details were registered at hospital office and independent researcher assigned an identification number to each family.

### Measures

All parent and children included in the study were evaluated at baseline (T0) and after 6 months (T1) at the end of the interventions, by two independent raters, who were blinded of treatment allocation. They administered the gold-standard tools for the clinical diagnosis of ASD, such as ADOS-2 test. The Adaptive Behavior Assessment System II (ABAS-II) questionnaire was used to assess adaptive functioning. Furthermore, the non-verbal cognitive abilities were assessed only at baseline, during the clinical evaluation at the time of diagnosis, using the Brief IQ of the Leiter International Performance Scale—Revised (LEITER-3) (33). The Griffiths Developmental Scales (Griffiths) were chosen to assess younger patients or those with more severe cognitive disabilities, for whom standardized tests like the Leiter may not be suitable (34). The Socio-Economic Status (SES) was investigated only at baseline, using SES (35).

#### Primary outcome

The primary outcome measure was parent–child interaction assessed by the Parent Interactions with Children: Checklist of Observations Linked to Outcomes (PICCOLO Checklist) (36, 37).

The PICCOLO is an observational measure in which a parent–child interaction is video recorded and trained observers code-specific parenting behaviors known to predict children's early social, cognitive, and language development. Four domains were obtained from each observation: “emotional involvement”, “reactivity”, “encouragement” and “teaching”, in line with our primary outcome. Furthermore, a raw “total score” was signed for each participant by the raters.

The videos had duration of 45 min, and six different raters were involved at baseline and at T1. At each baseline and T1 visit, 45 min of interaction between the parent, child, and therapist were videotaped for each participant. Subsequently, for coding purposes, only a subset of recordings involving spontaneous and meaningful interactions between the child and parent, and vice-versa, were selected. These recordings could include different moments from the first session at baseline and from the last session at T1. They were then coded by each rater independently from the others, both at T0 and at T1. Each rater did not evaluate the same child across both visits, and they were blinded from the treatment of participants at baseline.

Each domain comprises seven to eight individual items that are rated on a 0–2 scale, where 0 = absent, 1 = rarely/briefly, and 2 = frequently. A total of 80 interactions were rated: 40 at T0 and 40 at T1. Mean scores for each domain (“emotional involvement”, “reactivity”, “encouragement” and “teaching”) were calculated at each designated time point and utilized in analyses. The PICCOLO was calibrated for children aged between 10 and 47 months of age. Around 40% of children who participated in this study were older than 48 months. As a result, PICCOLO raw scores, rather than standardized scores, were used to assess pre- and post-intervention parenting behaviors, in each of the four domains.

All coders met a reliability criterion of 80% agreement with coding keys across two separate video recordings prior to beginning independent coding.

Finally, the internal consistency Cronbach's alpha coefficient was assessed for the PICCOLO items.

#### Secondary outcomes

Concerning the secondary outcomes measures, ASD symptom severity was assessed by the ADOS-2 (38), administered by a blinded, well trained, and licensed clinical psychologist. The ADOS-2 is a semi-structured assessment of communication, social interaction, and restricted/repetitive behaviors for individuals suspected of having ASD. It includes five modules depending on developmental, age, and language levels. In this study, we used the ADOS Toddler module and ADOS-2 Module 1, Module 2 and Module 3. The Calibrated Severity Total Score (CSS) was calculated for each participant. The CSS Total ranges from 1 to 10 and makes it possible to compare different modules of ADOS-2 by controlling for participants' age and language levels (39).

To assess adaptive functioning, we used the Italian version of the ABAS-II (40). ABAS-II consists of eleven skill areas organized into three general domains: Conceptual Domain (CD), Practical Domain (PD), and Social Domain (SD), alongside a comprehensive score known as the General Adaptive Composite (GAC), which was calculated by summing up scaled scores from the 10 skill areas. For the analysis, composite scores from all adaptive domains (CD, PD, SD, and GAC), expressed as a mean of 100 with a standard deviation of 15, were utilized. These scores were evaluated based on the ABAS-II forms used for the assessment. Depending on their child's age, parents choose either the “0–5 years” form or the “5–21 years” form.

Parental stress level was evaluated by using the PSI-SF (41). The PSI-SF consists of three subscales, namely Parental Distress (PD), Parent–Child Dysfunctional Interaction (P-CDI), and Difficult Child (DC). The sum of the items allows the calculation of a total PSI total score converted in percentiles.

The socio-economical status was also investigated for all participants at baseline, using a parent self-report interview (35), which combines both educational and occupational items.

### Interventions

#### Treatment as usual control group (TAU CG)

The TAU provided at the Child Neuropsychiatry Units of Sardinia consisted of individual psychosocial interventions inspired by NDBI, delivered by trained therapists.

The intervention was administered for 3 h per week over a period of 6 months, with each session lasting approximately 45 min.

Therapists used a combination of behavioral and developmental strategies to promote key skills in children with autism. They focused on improving communication skills by encouraging functional communication, social interaction through eye contact and facial expressions, and language developmental. In term of social skills, therapist helped children understand turn taking and engage in reciprocal interactions. For cognitive skills, they incorporated activities like symbolic play, problem solving and imitation.

The TAU was delivered for 3 h per week for 6 months. Each session lasted approximately 45 min.

#### Cooperative parent-mediated therapy—CPMT

CPMT was implemented at the Child Neuropsychiatry Units in a community Health Care Service site in Sardinia (Italy), in line with the previous description of Valeri and colleagues (32).

The aim of CPMT was to improve parental skills, to enable parents to promote in their child the following eight target skills: socio-emotional engagement, emotional regulation, imitation, communication, ostensive communication, joint attention, play and cognitive flexibility, and cooperative interaction. For each target skill, an individualized treatment plan was designed for each child in order to determine his developmental level and treatment goals. To assess the current level of the child on each target skill and to program individualized short-term and long-term goals, therapist completes a checklist based on the eight target skills at the beginning of the intervention CPMT strategies used were live active coaching in association with live modeling, live and video feedback. The CPMT was performed in a dedicated playroom at the hospital; the setting was organized with toys suitable for each child's age range (toys different from those used for the assessment). Parents and their child followed the therapy for 6 months, for a total amount of 15 sessions of 60 min; twelve core sessions (one session per week) were delivered in the first 3 months, followed by 3 monthly booster sessions. Each weekly core session had a specific focus and specific intervention strategies based on active parent coaching during parent–child interaction, and included the parent–child dyad with the parent being actively coached by a trained therapist. Live active coaching increases parents' competence in implementing strategies to enhance child development, and at the same time increases their confidence that they are able to do so, following the caregiver capacity-building approach (42, 43). Regarding the parent–child interaction, the therapist coached parents in order to develop specific strategies related to the main topics of the session and provided live modeling and specific live feedbacks on the parents’ use of these strategies and the child's response, in order to promote and facilitate the child's acquisition of specific skills. Feedback was provided to the parents during parental–child interaction in each session and through video feedback in five specific sessions. At the end of each session, a memorandum on the specific topic was given to the parents and homework was assigned. Parents were required to work with the child for at least 1 h daily. All interactions between the parent, child and therapist were videotaped. During the first and last session, parent–child spontaneous interactions were video-recorded for future video coding. Parents were asked to engage in spontaneous play with their child using a specific set of toys different from those used in the sessions. Two clinical psychologists, specifically trained in intervention in ASD, administered the CPMT lessons.

#### CPMT training

Twenty-one therapists received specific training totaling 24 h over a 4-month period, along with 10 h of supervision conducted by an experienced CPMT trainer. The CPMT training included didactic lectures, discussions, and video reviews with live coaching. Therapists were introduced to the eight core competencies of CPMT, starting with identifying developmentally appropriate intervention goals. They learned to use live coaching and modeling strategies to promote these competencies. Following the training, therapists took a final exam. Those who passed applied CPMT in a single case, receiving an additional 10 h of group supervision before implementing CPMT with the children in our study.

### Statistical analysis

To estimate the sample size, mean values of pre- and post-PICCOLO response scores from the literature (44) were retrieved. A standard deviation of 0.80 was estimated to account for potential variability. Using an alpha level of 5% and a power of 80%, along with a 15% dropout rate, the sample size required for the study is 30 units. Sample characteristics were described using mean and standard deviation (SD) and absolute and relative (percentage) frequency for quantitative and qualitative variables, respectively. Baseline differences between experimental and control groups were evaluated using Pearson Chi- or Fisher exact tests for qualitative variables (socioeconomic status, ADOS-2 modules, language and cognitive level) and by independent Student's *t*-test or the Welch-Satterthwaite correction for *t*-test in the event of an unequal variance for quantitative ones. Additionally, effect size was calculated using Cohen's delta (*d*) statistics. Inter-rater agreement for the PICCOLO total score (at baseline and at T1) was evaluated using the Intraclass Correlation Coefficient (ICC) and reported with their 95% confidence intervals (CIs). Specifically, at each time point, a subset of videos was independently coded by six raters, to assess inter-rater reliability for the total PICCOLO score.

To evaluate the effectiveness of CPMT, a repeated measures analysis of covariance (RM-ANCOVA) was adopted, with study outcomes (PICCOLO, ADOS-2, ABAS II and PSI scales) as dependent variables, time (two levels: T0 and T1) as a within-subjects factor, and treatment (two levels: CPMT and TAU CG groups) as a between-subjects factor. Based on the non-homogeneity for age at baseline, the inclusion in the model as a covariate was needed. In case of significant treatment × time interaction, the two within-treatment T0–T1 comparisons were performed, and a and a two-sided alpha level 0.05/2 = 0.025 was chosen as significant threshold. For other analyses, a *p*-value less than 0.05 were considered statistically significant.

Statistical analyses were conducted using STATA®17 (StataCorp, College Station, TX, USA).

## Results

At the baseline assessment the two groups (CPMT group and TAU CG) were homogeneously distributed for SES, language and cognitive levels (Table 1).

**Table 1:** Baseline characteristics of ASD children by study group.

Variable		*N* = 40	TAU CG (*n* = 19)	CPMT group (*n* = 21)	*p*-value
Socio-demographics					
Mean (DS) age (years)		5.1 (2.2)	6.0 (2.0)	4.3 (2.0)	0.02
Socio-economical status, *n* (%)	0	3 (7.7)	2 (10.52)	1 (4.76)	0.81
	1	10 (25)	5 (26.31)	5 (23.8)	
	2	10 (25)	5 (26.31)	5 (23.8)	
	3	11 (27.5)	5 (26.31)	6 (28.6)	
	4	5 (12.5)	1 (5.2)	4 (19.04)	
ADOS-2 modules					
*n* (%)	Toddler	1 (2.5)	1 (5.3)	0 (0.0)	0.51
	Module 1	9 (22.5)	6 (31.6)	3 (14.3)	
	Module 2	16 (40.0)	7 (36.8)	9 (42.9)	
	Module 3	12 (30.0)	4 (21.1)	8 (38.1)	
Language level, non-verbal patients *n* (%)		10 (25)	7 (36.8)	3 (14.2)	0.29
Non-verbal Brief Cognitive level (Leiter), IQ or Developmental Quotient (Griffith's) <70 *n* (%)		13 (32.5)	4 (21.05)	9 (42.9)	0.17

We show that at the baseline assessment the two groups (CPMT group and TAU CG) were homogeneously distributed for SES, language and cognitive levels, but not for age. TAU CG, treatment as usual control group; CPMT, cooperative parent-mediated therapy; ADOS-2, autism diagnostic observation schedule generic-second edition; IQ, intelligence quotient.

Nineteen participants were allocated to the TAU CG and 21 to the CPMT group. The sociodemographic characteristics at baseline assessment are presented in Table 1: the two groups were homogeneously distributed for socioeconomic status, ADOS-2 modules, language level, and cognitive level.

The results did not showed significant differences between the TAU CG and the CPMT group across multiple domains at baseline (Table 2). About post-treatment assessments, row and mean results are reported both for the TAU CG as well as for the CPMT group in the Table 3 below.

**Table 2:** Descriptive statistics for baseline outcomes by groups.

Primary and secondary outcomes	Baseline		
	TAU CG (*n* = 19)	CPMT group (*n* = 21)	*p*-value
	Mean (SD)	Mean (SD)	
PICCOLO			
Emotional involvement	9.7 (3.0)	10.2 (2.8)	0.64
Responsiveness	7.9 (2.1)	8.7 (3.0)	0.38
Encouragement	6.9 (2.1)	8.9 (2.6)	0.04
Teaching	6.1 (3.4)	7.2 (3.7)	0.37
ADOS-2			
Social affect	12.3 (4.4)	13.3 (3.3)	0.42
Restricted and repetitive behaviors	3.8 (2.0)	5.0 (2.5)	0.14
Total score	15.9 (5.0)	18.2 (4.0)	0.11
Comparison score	7.2 (1.7)	7.2 (2.1)	0.98
ABAS II			
Conceptual domain	66.3 (17.0)	67.0 (15.1)	0.90
Social domain	68.1 (17.3)	62.6 (13.3)	0.31
Pratical domain	64.0 (14.2)	62.5 (12.8)	0.75
General adaptive composite	64.7 (15.8)	63.3 (14.7)	0.79
Parental stress index (PSI)			
Parental distress	35.1 (13.4)	30.1 (12.8)	0.27
Parent–child dysfunctional interaction	30.7 (14.7)	26.4 (8.8)	0.33
Difficult child	34.1 (10.4)	33.0 (10.7)	0.75
Parents defensive response	20.4 (12.4)	16.8 (5.5)	0.31
PSI total score	73.3 (17.8)	69.5 (18.8)	0.55

Results of autistic symptomatology (ADOS-2), adaptive behaviors assessment scale (ABAS II) and parental stress index (PSI) outcomes at baseline (T0). We showed that there was not a significant increase between of PICCOLO scale scores, ADOS-2, ABAS II nor PSI for each domain at baseline (T0) in the two groups. TAU CG, treatment as usual control group; CPMT, cooperative parent-mediated therapy; ADOS-2, autism diagnostic observation schedule generic-second edition; ABAS II, adaptive behaviors assessment scale-second edition.

**Table 3:** Descriptive statistics for post-treatment outcomes by groups.

Primary and secondary outcomes	Post-treatment		
	TAU CG (*n* = 19)	CPMT group (*n* = 21)	*p*-value
	Mean (SD)	Mean (SD)	
PICCOLO			
Emotional involvement	10.8 (2.3)	12.6 (2.0)	0.01
Responsiveness	8.1 (2.6)	12.0 (2.1)	<0.0001
Encouragement	7.6 (2.2)	11.5 (2.8)	0.0001
Teaching	6.8 (3.4)	11.2 (3.4)	0.001
ADOS-2			
Social affect	12.3 (3.4)	10.4 (2.5)	0.10
Restricted and repetitive behaviors	3.7 (1.6)	2.6 (1.6)	0.09
Total score	15.9 (4.3)	13.1 (3.3)	0.048
Comparison score	7.3 (4.8)	4.8 (1.6)	<0.0001
ABAS II			
Conceptual domain	63.4 (18.0)	74.2 (23.5)	0.15
Social domain	62.3 (13.3)	74.9 (21.2)	0.09
Pratical domain	57.3 (16.4)	69.5 (19.6)	0.04
General adaptive composite	56.4 (16.4)	74.8 (24.6)	0.02
Parental stress index (PSI)			
Parental distress	29.3 (12.8)	23.4 (8.8)	0.18
Parent–child dysfunctional interaction	26.9 (10.8)	18.9 (6.5)	0.02
Difficult child	36.0 (11.8)	27.3 (8.8)	0.02
Parents defensive response	17.9 (7.1)	14.2 (4.9)	0.14
Parent stress index—total score	74.1 (22.3)	56.0 (17.9)	0.01

Results of autistic symptomatology (ADOS-2), adaptive behaviour assessment scale (ABAS II) and parental stress index (PSI) outcomes at T1. The CPMT experimental group showed a lower scores of ADOS-2 and of PSI-SF scores at T1 compared with the TAU control group at T1. Conversely, we report the higher ABAS II scores for the “social domain” and the overall score, compared to the TAU CG. TAU CG, treatment as usual control group; CPMT, cooperative parent-mediated therapy; ADOS-2, autism diagnostic observation schedule generic-second edition; ABAS II, adaptive behaviors assessment scale-second edition.

### Primary outcome: quality of parent-child interactions—(PICCOLO scale)

According to RM-ANCOVA, significant group × time interactions were found for responsiveness, encouragement, and teaching (Table 4). The model shows that decreases of subscales in the CPMT group was more relevant than those observed in the TAU CG. Specifically, Bonferroni-adjusted *post hoc* comparisons showed that the T0–T1 change in the CPMT group was significant for all subscales, whereas the TAU CG showed no changes (Table 4).

**Table 4:** Outcomes measures by groups.

Primary and secondary outcome measures		CPMT change T0–T1 (95% CI)	*p*	ES (Cohen's delta)	TAU change T0–T1 (95% CI)	*p*	ES (Cohen's delta)	RM-ANCOVA	
								Treatment × time interaction	
								Test	*p*
PICCOLO	Emotional involvement	−2.4 (−3.6; −1.2)	0.0004	−0.96	−1.1 (−2.7; 0.2)	0.08	−0.48	*F*_(1,34)_ = 1.80	0.188
	Responsiveness	−3.4 (−4.8; −1.9)	0.0001	−1.06	−0.4 (−1.7; 0.9)	0.55	−0.15	*F*_(1,34)_ = 9.54	0.004
	Encouragement	−2.9 (−4.5; −1.3)	0.001	−0.84	−0.6 (−2.0; 0.8)	0.36	−0.24	*F*_(1,34)_ = 4.72	0.037
	Teaching	−3.9 (−5.6; −2.3)	0.0001	−1.10	−0.9 (−2.9; 1.2)	0.38	−0.23	*F*_(1,34)_ = 6.12	0.019
ADOS-2	Social affect	2.9 (1.6–4.1)	0.0001	1.10	0.4 (−1.7; 2.5)	0.69	0.11	*F*_(1,33)_ = 5.17	0.029
	Restricted and repetitive behaviors	2.4 (1.4–3.4)	0.0001	1.12	0.6 (−0.7; 1.9)	0.33	0.26	*F*_(1,33)_ = 5.64	0.024
	Total score	5.3 (4.0–6.6)	<0.0001	1.40	0.7 (−1.4; 2.9)	0.48	−0.10	*F*_(1,33)_ = 15.91	0.0003
	Comparison score	2.4 (1.7–3.1)	<0.0001	1.56	−0.2 (−0.9; 0.5)	0.57	−0.15	*F*_(1,34)_ = 29.22	<0.0001
ABAS-II	Conceptual domain	−7.3 (−15.5; 1.0)	0.08	−0.42	2.9 (−7.2; 12.9)	0.55	0.33	*F*_(1,32)_ = 2.78	0.105
	Social domain	−11.6 (−20.0; −3.1)	0.01	−0.66	5.8 (−3.9; 15.5)	0.22	0.33	*F*_(1,32)_ = 8.22	0.007
	Pratical domain	−6.5 (−16.0; 3.0)	0.17	−0.33	6.7 (−3.0; 16.5)	0.16	0.38	*F*_(1,32)_ = 4.12	0.051
	General adaptive composite	−11.8 (−22.1; −1.5)	0.03	−0.55	8.3 (0.20–16.3)	0.045	0.57	*F*_(1,32)_ = 9.61	0.004
PSI	Parental distress	6.8 (2.4–11.1)	0.005	0.72	5.8 (−0.2; 11.8)	0.056	0.54	*F*_(1,33)_ = 0.08	0.78
	Parent–child dysfunctional interaction	7.6 (3.3–11.9)	0.001	0.83	3.8 (−4.2; 11.8)	0.33	0.26	*F*_(1,33)_ = 0.90	0.349
	Difficult child	5.7 (3.5–7.9)	<0.0001	1.20	−1.9 (−8.5; 4.8)	0.56	−0.16	*F*_(1,33)_ = 6.57	0.015
	Parents defensive response	2.7 (0.5–4.8)	0.018	0.58	2.5 (−3.6; 8.6)	0.40	0.22	*F*_(1,33)_ = 0.01	0.947
	Parent stress index—total score	13.5 (8.7–18.3)	<0.0001	1.33	−0.9 (−11.2; 9.4)	0.86	−0.05	*F*_(1,33)_ = 8.58	0.006

Presents the results of the repeated measures-ANCOVA (RM-ANCOVA) analysis for primary and secondary outcome measures, comparing score changes between the CPMT group and TAU CG from T0 to T1. The model was adjusted for the age of participants and evaluated the interactions between treatment and time. Within-group changes from T0 to T1 are reported with 95% confidence intervals (95% CI) and *p*-values. Bonferroni adjustment was applied for multiple comparisons. Effect sizes are presented as Cohen's delta (ES). CPMT, cooperative parent-mediated therapy; TAU CG, treatment as usual control group; ADOS-2, autism diagnostic observation schedule generic-second edition; ABAS II, adaptive behaviors assessment scale-second edition; PSI, parental stress index.

About the agreement between raters of the videos, ICC values suggest a high inter-rater agreement for the PICCOLO total score, both at baseline [ICC (95% CI): 0.97 (0.97–0.99); *p* < 0.001] and at T1 [ICC (95% CI): 0.98 (0.96–0.99); *p* < 0.001].

### Secondary outcomes: ASD core symptoms (ADOS-2), adaptive functioning (ABAS-II) and parental stress perceived (PSI)

The ADOS-2 scores reveal differences in autism symptom severity, particularly in social affect and restricted/repetitive behaviors. These findings may indicate a potential effect of CPMT on improving social communication skills and reducing repetitive or restrictive behaviors in children. The observed changes in ADOS-2 scores suggest that targeted intervention strategies may contribute to measurable improvements in core autism-related behaviors (Table 4). Detailed, significant group × time interactions were found for all ADOS-2 domains. Increases in the CPMT group were significantly higher than those of the TAU group. After, Bonferroni-adjusted *post hoc* comparisons T0–T1 change in the CPMT group was significant for all subscales; no significant changes were found in the TAU CG.

Regarding adaptive functioning, the ABAS-II scores highlight variations in conceptual, social, and practical domains (Table 4). In detail significant group × time interactions were observed for SD and GAC. After, *post hoc* comparisons T0–T1 change in the CPMT group was significant only for SAD, whereas no significant changes were found for the TAU CG.

The CPMT group appears to show improvements in daily living skills, communication abilities, and socialization compared to the TAU CG. These results suggest that CPMT may support the development of functional abilities essential for independent living and social participation.

Furthermore, the Parental Stress Index results indicate differences in stress levels between groups, particularly in parental distress and parent-child dysfunctional interaction subscales (Table 4). The reduction in stress levels in the CPMT group suggests a potential moderating effect of the intervention on caregiver burden, possibly due to improved child behavior and parental coping strategies. Significant group × time interactions were found for the difficult child subscale and PSI total score. After Bonferroni-adjusted *post hoc* comparisons the T0–T1 change in the CPMT group was significant for the difficult child subscale. No significant changes in the TAU CG.

Overall, these findings suggest that CPMT may have a beneficial impact on parent-child interactions, adaptive behavior development, autism symptom severity, and parental stress levels. While the observed differences highlight the potential efficacy of CPMT, further research is warranted to confirm these findings in larger and more diverse samples, exploring long-term effects and potential mechanisms underlying the observed change.

## Discussion

The current Clinical Experimental Study, with a random allocation design provides evidence for the effectiveness of a parent-mediated intervention (PMI) for children with ASD within a Community Health Service setting in Italy.

Our results suggest that the CPMT combined with TAU, even when delivered within a Community Health Service setting, may offer additional benefits for both parents and children with ASD compared to an individual TAU CG. Specifically, participants in the CPMT group exhibited improvements in parent-child interactions, a reduction in ASD symptom severity, enhancements in adaptive functioning, and a decrease in parental stress levels. Concerning the primary outcome, preliminary findings suggest that the addition of CPMT to TAU results in significant changes in the child's parental interaction style, in contrast to TAU alone, which did not involve parental engagement. Changes in parental interactions within the CPMT group were observed across all domains assessed by the PICCOLO Checklist. Notably, a robust effect was observed in the subscale of responsiveness, suggesting that a PMI intervention, when combined with TAU and implemented in community settings, can enhance caregivers' positive interactions with their children. In line with previous studies and consistent with another pilot PMI study showing significant improvements in positive parent-child interactions, as measured by the PICCOLO domains (44), our findings revealed significant improvements in parents' provision of encouragement and teaching opportunities, as well as in emotional involvement. This suggests that parents may be more motivated to foster positive affective connections, driven by the rewarding experience of achieving enhanced social communication with their child. This motivation could also be influenced by the improvement in their child's social communication, highlighting the potential bidirectionality of the effect. It is now known indeed that parents of children with ASD, although just as responsive as parents of typically developing children (45), have fewer opportunities to engage in adequate social responses due to the differences in the way their children learn from their environment (46). One possible explanation for this might rely in the difficulties parents face in distinguishing between functional directiveness, encouragement, and teaching, and overstimulation, especially with children who do not express their needs clearly. Several studies highlight that parenting interaction styles, such as responsive, directive, and encouraging, can influence joint engagement and promote the social and communicative skills of children with autism (47–50). A balance between directive and responsive strategies is therefore crucial to foster social and communicative development (51). Our study documented that CPMT efficacy extends to improving other dimensions of parent-child interactions beyond responsivity, such as strengthening encouragement and teaching domains. We could hypothesize that parents in the CPMT group, compared to parents of children in the TAU CG, employed a greater number of strategies, learned through live coaching, to promote the target competencies of CPMT. These competencies include, for instance, facilitating reciprocal social turns, using emotional regulation strategies, engaging in social imitation, and creating shared activities. The application of these strategies may have influenced the parents' ability to observe their child's behavior, provide feedback, encourage child' emotional expression, and respond flexibly to changes in their child's activities. In sum, our results confirm previous studies showing that PMIs are effective in improving parental global strategies in positive interactions (21, 52) and our findings are similar to those obtained in trials focused on community-based services that emphasize developmental, relationship-based, parent-mediated interventions for children with ASD (53). These encouraging findings offer preliminary support for the potential advantages of systematically integrating PMIs delivered into community service contexts. Regarding the secondary outcomes, our results suggest that the addition of CPMT to TAU, delivered within an Italian community health service, was effective in reducing ASD symptoms, improving adaptive behavior, and decreasing parental stress levels, in comparison to TAU alone. First, our results showed that after 6 months, at the end of the intervention, children with ASD in the experimental CPMT plus TAU CG exhibited a significant reduction in the severity of autistic symptoms, as measured by ADOS-2 scores, compared to those who received only individual TAU. This finding confirms and extends the results of a previous study in which CPMT, delivered in a research service setting, led to a significant short-term improvement in both social-communication skills and autistic symptoms in children with ASD (32).

While early meta-analyses on PMIs indicated short-term improvements in parent outcomes but no significant effects on child outcomes (4, 5), our findings align with more recent meta-analyses that report moderately strong improvements in child outcomes in favor of PMIs (6). The significant improvement in ASD core symptoms of the CPMT group, could be related to specific behavioral and developmental strategies focused on live active coaching, live modeling, live feedback, and video to promote target skills adapted to the child's developmental level.

Second, we found that CPMT, developed within a community-based setting, can also be effective in improving the adaptive skills of children with autism, at least in the short term. Specifically, our results show that children in the experimental CPMT plus TAU CG demonstrated significant improvements in the Practical Domain, Social Domain and General Adaptive Domain assed by the ABAS II questionnaire.

In both groups, no significant improvements were observed in the Conceptual Domain. However, differences in the social domain between baseline and T1 approached statistical significance when comparing social skills in the CPMT group, suggesting that CPMT may have a positive impact on the children's adaptive social skills as reported by parents.

Our results differ from recent findings that indicate a small effect in adaptive skills from PMIs, although, some studies documented improvements also in adaptive behaviors/life skills (6, 54).

Children in the TAU CG did not show significant improvements in adaptive skills. One possible explanation for this is that parents did not receive specific guidance or have an active role in the intervention, as they did in the experimental group. Research, in fact, suggests that including parents in interventions, particularly parent-mediated interventions, can have a positive impact on the abilities of children with autism (8, 55, 56).

Parents in the CPMT group actively participate in the intervention and, through therapist-guided coaching, learn to generalize skills to daily life. This active involvement may explain why children in the experimental group show improvements in adaptive functioning, in contrast to those in the individual therapy group, where parental involvement is either limited or not explicitly defined. Nonetheless, this result should be interpreted with caution, as adaptive functioning was measured using the ABAS, a self-report tool, and the parents were not blind to the intervention. Therefore, it may be useful for future studies to use a direct assessment tool for adaptive functioning, such as the Vineland scales.

Finally, our results suggest that CPMT could be effective in reducing parenting stress levels after 6 months, at the end of intervention. This result is consistent with previous literature which has documented significant improvement in parenting stress levels after PMI delivery (24, 32, 57). Some putative mechanisms explaining the benefits of CPMT on parenting stress can be identified.

The better parent-child interactions might have positive effects on parents' self-efficacy on parenting domain with a “cascade” effects on stress levels (58). In the CPMT group, the use of real-time active coaching, combined with strategies aimed at enhancing caregiver skills shows promising results (59, 60); however, this association requires further validation (61, 62). Despite the promising results from the questionnaires, these findings should be interpreted with caution, as the informant was not blinded.

Altogether, these promising results provide preliminary for the potential benefits of integrating CPMT into community health services through a research-community partnership.

However, several limitations of the present study must be discussed. The limited sample size restricts the generalizability of the results. Further studies with larger sample sizes are needed to confirm these findings. Additionally, no systematic methods were employed to assess treatment fidelity. Another limitation is that the parent who reported on their stress and adaptive functioning were not blinded to their child's intervention group allocation, which could have introduced bias.

Despite these limitations, the current study provided new insights into the efficacy of CPMT on parent-child interaction, by assessing it through a clinician-based instrument. Our study shows that a specific targeted PMI, namely the CPMT, could lead to improvement in ASD core symptoms, adaptive functioning and parenting stress levels. However, assessing the influence of cognitive abilities on CPMT efficacy will be a future research question to address in the continuation of the present study.

Furthermore, in our study design, we contemplated to use direct observational measures, and the clinical researchers who assessed the participants were blinded. Finally, CPMT conducted in a Tertiary Care Center (32) seems to show efficacy outcomes also in a community child neuropsychiatry service. Future studies aiming at the evaluation of the stability of the effects of CPMT in public community services, using a long-term follow-up design are strongly encouraged.

## Data Availability

The data analyzed in this study is subject to the following licenses/restrictions: the dataset contains sensitive or proprietary information and is therefore not publicly available. Access may be granted on a case-by-case basis for research purposes, pending approval from the relevant data governance authority. Requests to access these datasets should be directed to alessandra.carta@aouss.it.
